# Blast-induced axonal degeneration in the rat cerebellum in the absence of head movement

**DOI:** 10.1038/s41598-021-03744-4

**Published:** 2022-01-07

**Authors:** Robin Bishop, Seok Joon Won, Karen-Amanda Irvine, Jayinee Basu, Eric S. Rome, Raymond A. Swanson

**Affiliations:** 1 Department of Neurology, University of California at San Francisco, San Francisco, CA 94158 USA; 2 (127)Neurology, San Francisco Veterans Affairs Medical Center, San Francisco, CA 94121 USA; 3 Present Address: Anesthesiology Service, Veterans Affairs Palo Alto Health Care System, 3801 Miranda Ave (E4-220), Palo Alto, CA 94304 USA; 4 Present Address: Department of Anesthesiology, Perioperative and Pain Medicine, Stanford University, School of Medicine, Stanford, CA 94305 USA

**Keywords:** Neuroscience, Neurology

## Abstract

Blast exposure can injure brain by multiple mechanisms, and injury attributable to direct effects of the blast wave itself have been difficult to distinguish from that caused by rapid head displacement and other secondary processes. To resolve this issue, we used a rat model of blast exposure in which head movement was either strictly prevented or permitted in the lateral plane. Blast was found to produce axonal injury even with strict prevention of head movement. This axonal injury was restricted to the cerebellum, with the exception of injury in visual tracts secondary to ocular trauma. The cerebellar axonal injury was increased in rats in which blast-induced head movement was permitted, but the pattern of injury was unchanged. These findings support the contentions that blast per se, independent of head movement, is sufficient to induce axonal injury, and that axons in cerebellar white matter are particularly vulnerable to direct blast-induced injury.

## Introduction

An explosive blast produces an immediate increase in local air pressure that propagates outward from its source. The leading edge of this high pressure wave is followed within milliseconds by a negative pressure, and subsequently by a strong “blast wind” that can displace objects and people at high velocity. Individuals exposed to an explosive blast can thus suffer brain injury through multiple mechanisms, including penetrating skull injuries, rapid acceleration/deceleration head movements with shearing of vessels or nerve processes, rapid increase in intracranial pressure (transmitted from the thorax), and effects of the blast pressure wave as it passes through brain^1^. Brain injuries result from combinations of these factors, in addition to systemic factors such as shock and infection^2–4^, and it has thus been difficult to parse out what injury is be attributable specifically to effects of the blast wave itself.

Some studies suggest that mechanical effects of the blast wave per se on brain parenchyma—specifically axons, capillaries and venules—significantly contribute to brain injury from blast exposure^3,5–8^, but others have argued that direct effects of blast are minimal relative to the effects of head movement and resultant brain–skull collision and shear stress on brain parenchyma^3,9–11^. This question can, in principle, be resolved using a blast exposure with head movement strictly prevented and body shielded, but this requires careful control and assessment of head movement, particularly rotational head movement, as even limited head rotation can itself cause brain injury at high rates of acceleration^12,13^.

We addressed this question using a rat model of blast exposure in which the head was placed orthogonal to a blast tube, either fixed tightly in place or with lateral movement permitted during blast exposure. Head movement was assessed using high-speed video recordings, both to document lack of head movement in the head—fixed configuration and to quantify head acceleration rates where lateral head movement was permitted. Histological assessment of brains exposed to blast showed scattered axonal injury selectively in the cerebellar white matter, even with head movement strictly prevented. Forebrain white matter show injury was restricted to the optic nerve ipsilateral to the blast and its visual tract projections.

## Materials and methods

### Blast simulator shock tube

**Figure 1:**
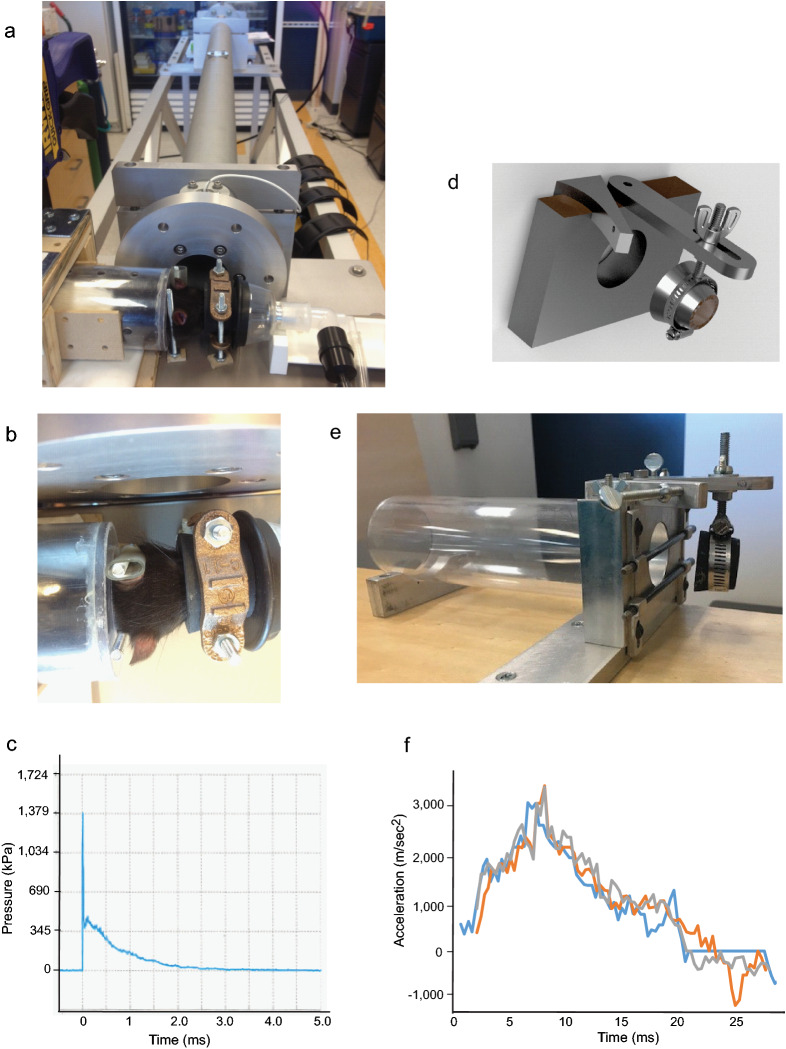
Blast simulator tube with head-fixed and head-movement rat holsters. (**a**, **b**) Blast simulator tube with rat in head-fixed configuration, from side and from top. (**c**) Trace of blast overpressure wave measured 2.54 cm from the tube orifice. (**d**, **e**) Schematic rendering and photograph of head-movement rat holster. Metal stop to the right of the fulcrum serves to prevent extreme neck flexion. (**f**) Three superimposed recordings of rat head lateral acceleration during blast exposure with rats placed in the head-movement holster.

A blast simulator shock tube was custom‐built by L-3 Applied Technologies Inc. (San Diego, CA). The apparatus consisted of a 4.5″ (11.43 cm) long compression chamber and a 65″ (165 cm) long × 2″ (5.1 cm) inner diameter expansion section (Fig. 1) that were separated by replaceable 0.005″ (12.7 mm) Mylar diaphragms (Hi-Tech Products). Shock waves were generated by filling the compression chamber with helium until rupture of the Mylar diaphragms, which released high-pressure gas into the expansion section. Use of three stacked diaphragms (38.1 mm total thickness) produced a blast with peak reflective pressure of (1380 ± 110 kPa) as measured 2.54 cm from the tube exit. Reproducibility of blast waves was monitored with in-tube incident blast wave pressure readings at each use. The shock wave was directed through the tube toward the head of a rat that was either held stationary or allowed to move laterally (Fig. 1). As endorsed by Smith and colleagues^14^, additional data elements pertaining to the blast tube and blast kinetics are provided in Table 1.

**Table 1:** Blast simulator tube characteristics.

Blast tube inner diameter	2 inches (5.1 cm)
Expansion tube length	65 inches (165 cm)
Compression chamber length	4.5 inches (11.4 cm)
Compression chamber peak pressure	3170 ± 209 kPa
Compression gas	Helium
Mylar burst membranes	12.7 mm (total thickness)
Tube incident pressure	483 ± 55 kPa (measured in tube, 7.62 cm from exit)
Reflected pressure	1380 ± 110 kPa (measured 2.54 cm from tube exit)
Distance from tube exit to rat head	2.54 cm
Animal shielding	Body shielded by plastic cylinder

Compression chamber pressure and tube incident pressure were monitored with each use, and values are means ± s.e.m. from 8 trials.

Reflected pressure is mean ± s.e.m. from 3 measures obtained during instrument calibration with compression chamber peak pressure 3170 kPa and mean tube incident pressure 483 psi.

### Blast exposure

Studies were approved by the San Francisco Veterans Affairs Medical Center animal studies committee and performed in adherence to the *Guide to the Care and Use of Experimental Animals* and *The Ethics of Animal Experimentation* and the 2020 ARRIVE guidelines^15^. Adult male Long Evans rats, age 3–4 months, were acquired from Simonsen Laboratories (Simonsen, CA) and acclimated for at least 1 week prior to exposure. The animals were kept on a 12 h light/dark cycle and fed ad libitum.

Six rats were used for an initial measurement of head movement in head-fixed condition, and 3 rats were used for determination of the lateral acceleration in the “head movement” blast condition. Thirty-eight rats were arbitrarily assigned to one of three experimental groups at the time of blast exposure: blast with head fixed, blast with head movement, or sham blast. Two of the rats died as a result of anesthesia complications at the time of blast exposure; one in the sham blast group and one in the blast with head movement group. These rats were replaced to provide an n of at least 3 for each blast exposure type and time point evaluated.

Male rats, 350–400 g were anaesthetized with 5% isoflurane in a closed induction chamber. Anesthesia was maintained with 2.5% isoflurane via a nose cone, and rectal temperature and tail blood pressure were monitored. For the head-fixed blast studies, the animal head was fixed in a stabilized rubber nose cone and positioned 2.54 cm from the blast tube orifice (Fig. 1). For the head-movement studies, the rat head was instead secured in a custom holder (HR Machinery, Fremont, CA) by snugging the snout into a rubber nose cone fixed to an overhead lever that allowed free lateral movement of the head up to 45° from neutral position (Fig. 1d). Movement beyond this range was prevented to eliminate the possibility of neck trauma. For both models, blast waves were delivered to the left side of the rat head, with the rat bodies shielded to prevent lung injury and pressure wave transmission from the thorax to brain^16^. Immediately following blast exposure, experimenters visually confirmed that there was no detectable head rotation or other displacement of the rat head in the holder. Anesthesia was discontinued and the rat was placed in a recovery cage for observation until ambulatory. Acetaminophen (2.7 mg/mL) was provided an analgesic to all rats (including those exposed to sham blast) in drinking water for 3 days post injury, as recommended by AAALAC guidelines. Brains were harvested at 1, 3, 7, or 30 days post-injury for histology studies. Rats undergoing sham blast exposure were treated identically to blast-exposed rats except that they were positioned above the blast tube, such that these rats experienced anesthesia and blast noise but no blast wave or wind.

Head movement in the head-fixed and head-movement blast exposures was quantified in an initial group of rats using a Phantom M-310 high speed camera recording 3200 frames per second (Vision Research, Wayne, NJ). The videos were replayed in stop-frame mode to permit measurement of linear displacement of the mobile fulcrum to which the rat head was fixed during each frame interval of 312.5 microseconds (Supplementary Video S1). The fulcrum point used for the measurements corresponded to the approximate location of the rat skull bregma. Velocity was calculated as linear distance moved/time, and acceleration at each time point was calculated as the change in velocity measured in consecutive 312.5 microsecond epochs. The raw data were smoothed by averaging the acceleration values over every 5 consecutive 312.5 microsecond observation epochs. In both the head-fixed and head-movement blast exposures, the direct observation and videography showed no rotational head movement. (Supplementary Video S2). Independent assessment showed that these observations were able to detect rotational displacement of 3 degrees or greater in 6 of 6 rats observed under conditions in which rotary head movement was allowed by loosening the nose cone.  

### Immunohistochemistry

Rats were anesthetized and transcardially perfused with 200 ml of cold 0.9% saline followed by perfusion with 4% paraformaldehyde solution in 0.1 M sodium phosphate buffer, pH 7.4, for 7 min. The harvested brains were post-fixed in the 4% paraformaldehyde/phosphate buffer solution for 24 h and then immersed in 20% sucrose for 2 days. Cryostat sections (40 μm thickness) were prepared through the forebrain and cerebellum.

For immunostaining, sections were incubated with blocking buffer (2% goat serum and 0.15% Triton X-100 in 0.1 M phosphate buffer) followed by the primary antibody overnight. The primary antibodies employed were goat anti-Iba1 (Abcam, Cambridge, MA; Cat #Ab107159), mouse anti-NF-H (BioLegend, San Diego, CA; Cat. #801601), rabbit anti-APP (Invitrogen, Carlsbad, CA; Cat #51-2700) and biotinylated goat anti-rat IgG (Vector laboratories, Burlingame, CA; Cat. #BA-1000).The sections were subsequently incubated with corresponding biotinylated anti- IgG secondary antibodies for 2 h (Vector laboratories, Burlingame, CA; Cat. #BA-2001 for mouse, #BA-9400 for rat, #BA-9500 for goat). Antibody binding was visualized with the diaminobenzadine method using a Vector Labs ABC kit. Photographs (510 µm × 380 µm) were taken in two sections from each hemisphere and structure of interest, as shown in the figures. Iba-1 expression and IgG extravasation were evaluated in sections adjacent to those used for silver staining. In each case, the NIH Image-J program was used to measure mean signal intensity X area of signal to calculate integrated signal intensity (I_integrated_). Calculations used the formula I_integrated_ = (I_measured_ − I _background_) × Area, with I_background_ determined from a region of each photo that was clearly devoid of either Iba-1 or IgG immunostaining, and Area defined as the number of pixels within each photograph with I_measured_ > I_background_. Both the photography and data analysis were performed by individuals who were blinded to the experimental conditions.

The modified Gallyas hydroxylamine silver protocol was used to detect degenerating axons^17,18^. (Note this method is different than the classical Palmgren silver stain used for general visualization of neuronal morphology.) Brain sections mounted on slides were post-fixed in 4% paraformaldehyde solution in 0.1 M sodium cacodylate buffer for 1 h, then incubated with 0.6% ammonium nitrate in 4.5% sodium hydroxide for 10 min. The sections were then incubated with 10% silver nitrate and 6.4% ammonium nitrate in 5.4% sodium hydroxide for 20 min and washed in saline. After final washing with 0.5% acetic acid, the sections were dried and cover-slipped. For double labeling with Iba-1, the immunostaining was performed first and silver staining second. To quantify silver-stained neurites, 510 µm × 380 µm photographs were taken were taken in two sections from each hemisphere and structure of interest, as shown in the figures. The number of independent (non-colinear) silver-stained fibers per photograph was manually counted in each photograph by an observer who was blinded to the experimental conditions.

For identifying degenerating neuronal cell bodies in the brain, fluoro-jade B staining was performed as described previously^19,20^. Photographs were taken at pre-determined bilateral regions in two sections from the cerebral cortex, hippocampal CA1, cerebellar cortex, and cerebellar white matter from each animal. Data were quantified by manual counting of fluoro-jade B positive cells per photograph.

### Statistics

Results were assessed in each brain region independently. Within each region, differences in silver staining ipsilateral vs contralateral to the blast exposure and between the observation time-points were compared by the Kruskal–Wallis and Mann–Whitney tests. Comparisons were made between the sham and blast conditions by after averaging the ipsilateral and contralateral values from each brain and combining all blast time-points. The Iba-1 expression data were analyzed by the same methods. Differences were considered significant at *p* < 0.05.

## Results

### Characterization of blast exposure

The blast wave peak pressure measured at the location of the rat head placement (2.54 cm from the blast tube orifice) was 1380 ± 110 kPa (Fig. 1c). Where lateral head movement was allowed, the peak lateral head acceleration was 3167 ± 133 m/s^2^ (Fig. 1f), which is comparable to that obtained in prior studies^21,22^. Other details of the blast generation and dynamics are provided in Table 1.

### Axonal injury and microglial activation in the cerebellum

Several brain regions were assessed in rats euthanized 1, 3, 7 and 30 days after blast exposure: cerebral cortex, striatum, hippocampal CA1, corpus callosum, lateral optic tracts, cerebellar cortex, and cerebellar white matter. None of these areas showed any definitive IgG staining (a marker of blood–brain barrier disruption) or fluoro-jade B staining (a marker of neuronal death) in either the head movement or head fixed conditions, at any of the time points evaluated (data not shown). Positive controls for each of these markers were obtained in a study of penetrating head trauma that was performed in parallel to this study and previously reported^23^.

**Figure 2:**
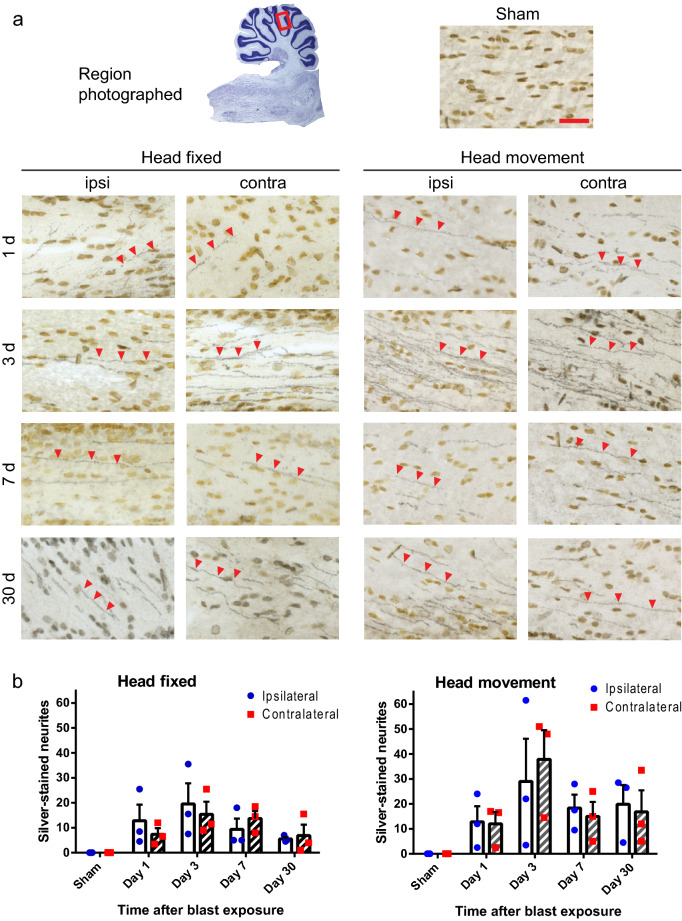
Injured nerve fibers in superficial cerebellar white matter. (**a**) Silver staining identifies injured nerve fibers (black, red arrowheads) and cell nuclei (brown) in sections taken ipsilateral and contralateral to blast impact. Heads were either fixed in place or allowed to move laterally during blast exposure, and brains were harvested at the indicated time points. Sections from control (sham blast exposure) rats showed no detectable silver staining. Scale bar = 30 µm. Images are representative of n = 3 rats treated under each condition and time point. (**b**) Quantification of silver-stained neurites (means ± s.e.m per field; n = 3). In both the head-fixed and head-movement conditions, the number of injured neurites was increased relative to the sham condition. (*p* < 0.01 by the Mann–Whitney test). Differences between the two sides and between the four time points assessed were not statistically different in either condition.

**Figure 3:**
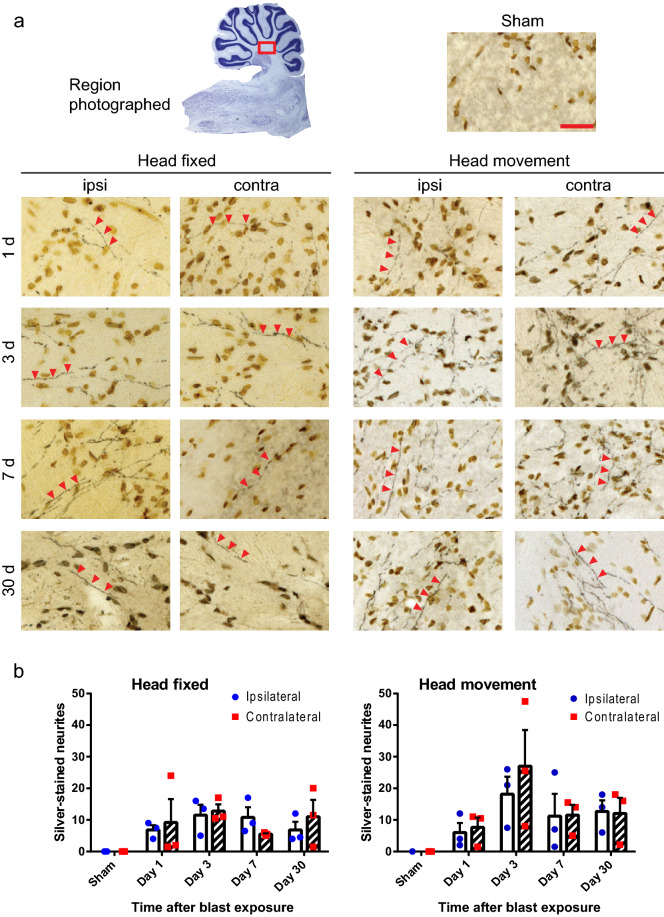
Injured nerve fibers in deep cerebellar white matter. (**a**) Silver staining identifies injured nerve fibers (black, red arrowheads) and cell nuclei (brown) in sections taken ipsilateral and contralateral to blast impact. Heads were either fixed in place or allowed to move laterally during blast exposure, and brains were harvested at the indicated time points. Sections from sham-treated rats showed no detectable silver staining. Scale bar = 30 µm. Images are representative of n = 3 rats treated under each condition at each time point. (**b**) Quantification of silver-stained neurites (means ± s.e.m per field; n = 3). In both the head-fixed and head-movement conditions, the number of injured neurites was increased relative to the sham condition. (*p* < 0.01 by the Mann–Whitney test). Differences between the two sides and between the four time points assessed were not statistically different in either condition.

By contrast, silver staining showed scattered axonal injury in the cerebellar superficial and deep white matter in both blast conditions (Figs. 2, 3). In the head-fixed condition, the silver staining was observed at day 1 after blast exposure and remained evident at 30 days. There was no discernible difference in the pattern or density of injured fibers observed in cerebellar regions ipsilateral versus contralateral to the blast impact. Rats exposed to blast with lateral head movement allowed showed the same pattern of cerebellar axonal injury. In both the superficial and deep white matter the density of stained fibers appeared greater at every time point in the head movement allowed condition, but this difference did not achieve statistical significance. Silver stained fibers were never observed in cerebella from the sham-injured rats, nor in the cortex, striatum, hippocampal CA1, or corpus callosum of the rats with positive signal in the cerebellum (Supplementary Figs. S1, S2).

**Figure 4:**
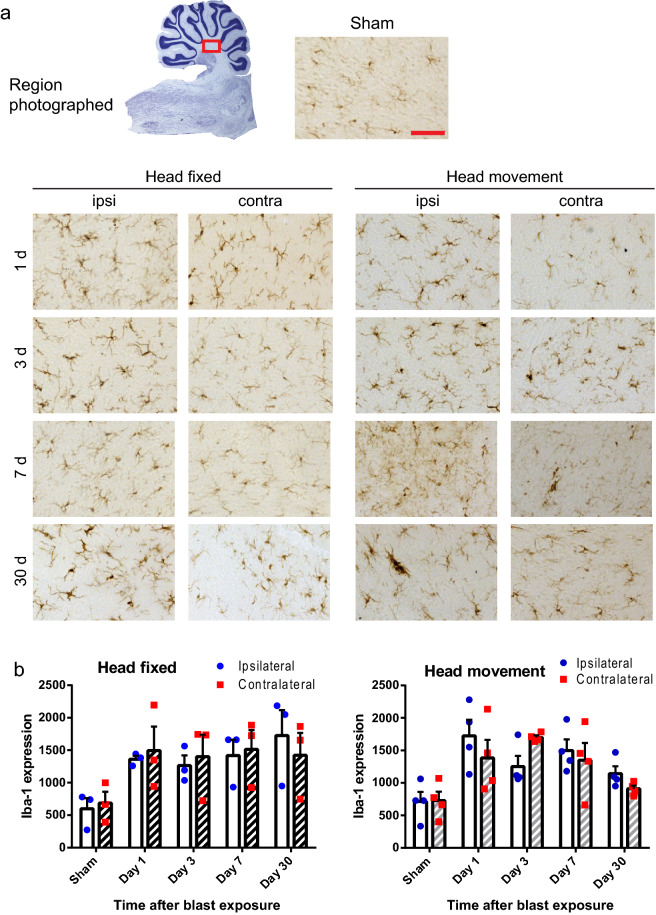
Microglial activation in cerebellar white matter. (**a**) Scattered microglia with activated, hypertrophied morphology and increased Iba-1 immunoreactivity (brown) are evident in brains from both the head-fixed and head-movement blast exposures at all time points evaluated. Scale bar = 30 µm. Images are from sections taken ipsilateral and contralateral to blast impact and are representative of n = 3 rats in each condition and time point. (**b**) Quantification of Iba-1 expression, means ± s.e.m; n = 3–4). In both the head-fixed and head-movement conditions, Iba-1 expression was increased relative to the sham condition. (*p* < 0.05 by the Mann–Whitney test). Differences between the two sides and between the four time points assessed were not statistically different in either condition.

**Figure 5:**
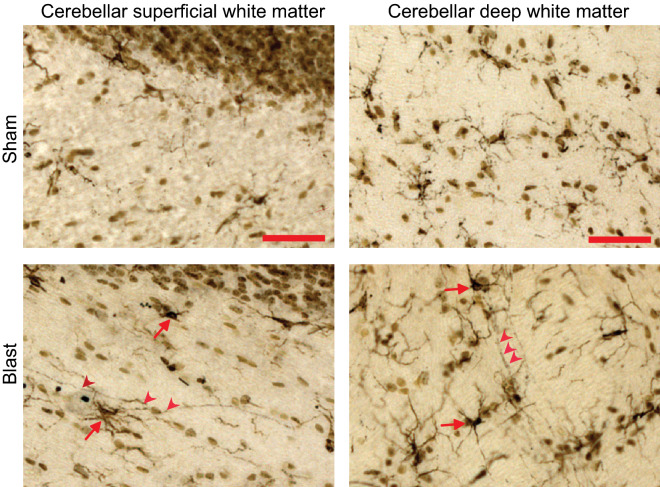
Microglial association with injured axons. High power views of cerebellar white matter show Iba-1 positive microglia with activated, hypertrophied morphology (brown, red arrows) are in contact with silver-stained fibers (black, red arrowheads). Images are from brains harvested 7 days after exposure to blast with heads fixed in place. Scale bar = 50 µm.

Immunostaining with the microglial marker, Iba-1, revealed cells with an enlarged cell soma and shortened processes indicative of an activated morphology within the cerebellar white matter of both blast exposed groups (head-fixed and head-movement) when compared to sham injured rats (Fig. 4). High magnification images revealed that the hypertrophied microglia were frequently in contact with silver-stained axonal processes (Fig. 5).

**Figure 6:**
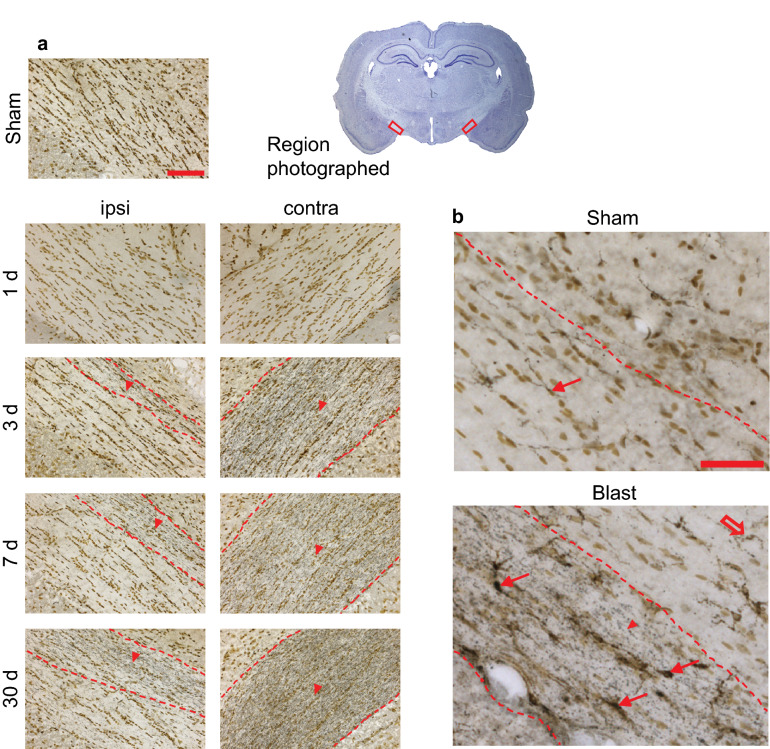
Degenerating nerve fibers in optic tracts. (**a**) Silver staining identifies degenerating nerve fibers (black, in regions marked by red arrowheads and dashed lines) and cell nuclei (brown). Note dense fiber degeneration contralateral to blast, scattered fiber degeneration ipsilateral to last, and none in sham-treated rat brain. Scale bar = 100 µm. Images are representative of n = 3 rats treated at each time point and condition. (**b**) Double labeling for microglia (red arrows) and silver-stained fibers (arrowheads) in a section taken contralateral to blast impact, 7 days after blast. Note microglia outside the optic tract marked by dotted lines do not exhibit activated morphology (open red arrow). Scale bar = 50 µm.

The silver stained axons occasionally showed focal varicosities, most easily discerned with high power views (Fig. 6). Immunostaining for neurofilament-H was performed to more generally show axonal morphology, and this also showed scattered varicosities in the cerebellar white matter of blast exposed rats. Axons in the cerebellar white matter also showed evidence of focal amyloid precursor protein (APP) accumulation, which is a marker of disrupted axonal transport (Supplementary Fig. S3). The axonal varicosities and APP accumulations were observed only in the cerebellar white matter of blast-exposed rats, and not in control rats or other brain regions, other than optic tracts as noted below.

### Fiber degeneration in the visual system

**Figure 7:**
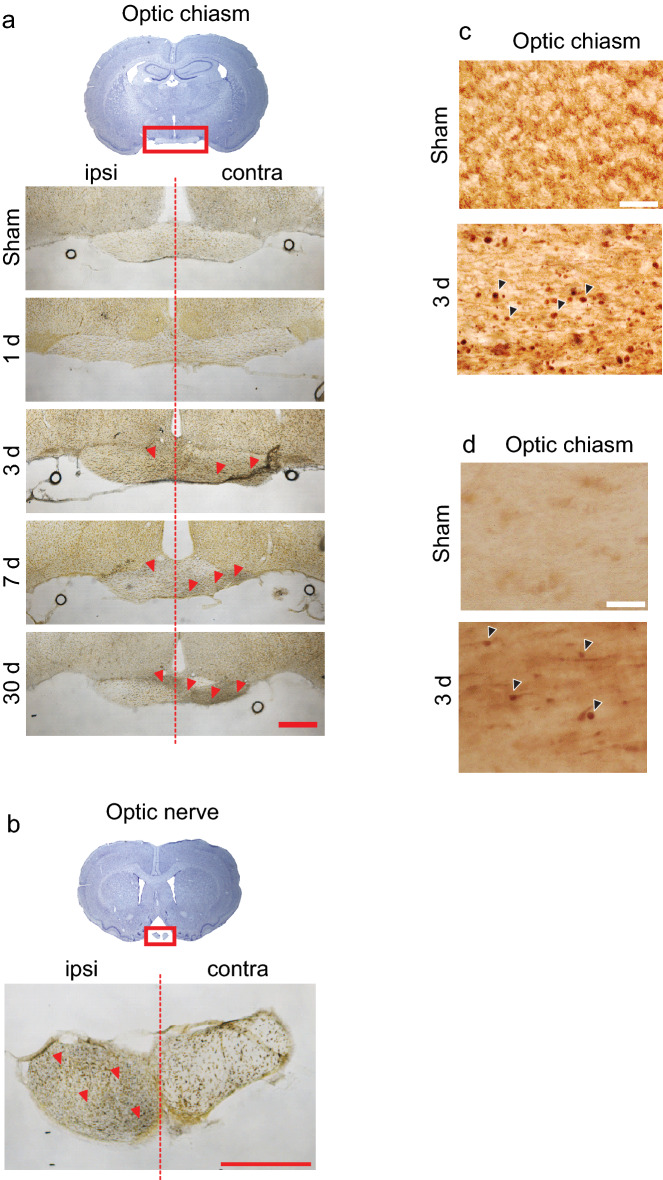
Degenerating fibers in optic nerve and optic chiasm. (**a**) Silver staining identifies degenerating nerve fibers (black) and cell nuclei (brown) in the optic chiasm of brains harvested at the indicated time points after head-fixed blast exposure. Red arrowheads show areas containing the very fine silver-stained processes. Representative of n = 3. Scale bar = 500 µm. (**b**) Silver stained fibers in the optic nerve were present exclusively in the nerve ipsilateral to blast (representative of n = 4). Note that fibers in these sections are running orthogonal to the plane of section. Scale bar = 500 µm. (**c**) Blast-induced axonal varicosities in the optic chiasm as shown by immunostaining for neurofilament–H. (**d**) Blast induced foci of amyloid precursor protein in the optic chiasm. Representative of n = 3. Scale bar = 30 µm for (**c**) and (**d**).

In addition to the axonal injury in cerebellar white matter, axons in the optic nerve and visual tracts showed robust sliver staining after blast exposure; however, the time course and anatomical pattern observed suggested a process secondary to injury to the eye facing the blast wave, rather than a direct effect of blast on the brain parenchyma. Unlike in cerebellum, silver-stained fibers in the optic tracts were not detected until 3 days after blast, and became progressively more numerous at 7 and 30 days (Fig. 6). Moreover, the optic tract contralateral to the blast was affected to a far greater extent than the ipsilateral optic tract. Silver staining of the optic chiasm showed the same pattern as the optic tracts: a delayed, predominately contralateral fiber degeneration (Fig. 7). In some rats, a segment of the optic nerves remained attached to brain through the processes of brain harvest, fixation, and sectioning, and assessment of these nerves showed silver-stained fibers exclusively in the ipsilateral optic nerve (Fig. 7). These findings are consistent with the neuroanatomy of the rat visual system, in which about 90% of fibers from each optic nerve cross in the optic chiasm versus 50% in humans^24^. The axonal injury identified in the visual system was in other respects similar to that observed in the cerebellum; co-labeling for Iba-1 and silver staining showed hypertrophied microglia in contact with the silver-stained fibers, and immunostaining for neurofilament-H and APP showed axonal varicosities and foci of APP accumulation, respectively (Fig. 7).

## Discussion

There remains uncertainty as to what aspects of blast-induced brain injury can be attributable to direct effects of blast wave on brain tissue, as opposed to other events associated with blast exposure, such as rapid brain acceleration/deceleration and brain impact against the skull. To address this issue, we utilized a blast injury model in which the rat head was firmly fixed to prevent acceleration/deceleration forces, and the body shielded to prevent transmission of the pressure wave from thorax to brain. Axonal injury was observed even with no head movement, thus supporting the idea that axonal damage can be mediated by physical aspects of the blast wave per se. This axonal damage was limited to the white matter of the cerebellum and not detected in any forebrain structures other than the visual tracts, in which axonal degeneration was likely secondary to unilateral ocular trauma. This pattern suggests an inherently increased vulnerability of cerebellar white matter to direct blast-induced injury.

Axonal injury is a well-established consequence of blast exposure, but it remains unclear whether this injury is caused by direct physical effects of blast wave on axons or is instead entirely attributable to indirect effects of blast-induced head movement^14^. Most published studies of experimental blast injury do not detail methods to restrict head movement, and in those that do, the actual effectiveness of these efforts is not well documented. This may be a critical experimental variable because head movement alone, particularly rotational movement is sufficient to induce brain injury^25–27^. Indeed, sagittal rotations of less than 20 degrees in can produce widespread axonal injury^28^, and the minimal head movement required to cause axonal injury is not defined. A key aspect of the present report is that the “head-fixed” configuration prevented movement in any direction, and the effectiveness of this fixation was confirmed by videography.

Acute axonal injury comprises a spectrum of pathologies at the sub-cellular level that range from incomplete disruption of axonal cytoskeleton and impaired axonal transport to complete axotomy^29,30^, and this acute injury is compounded by a range of secondary injury and inflammatory process that may continue long after the initial insult^30–32^. Histological assessments of axonal injury are correspondingly varied, and include markers for axonal transport, morphology, and integrity. Gallyas silver staining as employed here is a highly sensitive indicator of axonal injury and is thought to reflect proteolytic exposure of normally inaccessible hydrophobic sequences that, when appropriately treated, become nucleation sites for silver aggregation^33,34^. This method has been widely used to identify traumatic axonal injury, though not always with other confirming markers. In the present study several features of the silver staining confirm that it is indeed identifying damaged axons: (1) silver stained axons were exclusively observed in the blast-exposed brains, and never in the sham (control) brains; (2) silver stained axons were frequently associated with hypertrophied microglia; (3) the silver stained axons occasionally exhibited varicosities, which are morphological hallmarks of axonal injury; (4) immunostaining the axonal cytoskeleton with neurofilament-H likewise identified axons with varicosities in the brain regions that silver staining axons were identified and not in other regions or control brains; and (5) foci of APP accumulation were also identified in the brain regions containing silver-stained axons and not in other regions or control brains. In our hands the silver staining technique provided a more robust signal than either neurofilament-H or APP immunostaining. This may reflect the intrinsic relative sensitivity of these methods to axonal injury, as also suggested by prior reports^35–37^.

Our findings were also notable for a lack of IgG extravasation (indicative of blood–brain barrier damage) or cell death in blast-exposed brains. This observation is generally consistent with prior reports of studies in which head movement was limited. Thompkins and colleagues, using a blast tube simulator similar to that used here with and head movement limited but not prevented, observed scattered foci of inflammatory cell infiltration and reactive astrocytes, but no cell death^38^. Yeoh et al.^39^, using blast exposures with significantly greater intensity than used here, identified scattered small areas of IgG extravasation in forebrain but no other reported pathology. In that study, the rat was offset from the tube orifice and videography showed little movement during blast exposure, similar to the present work. Other studies of blast injury similarly report negligible or no neuronal death, though scattered hemorrhages and biochemical and gene expression changes have been observed^6,40–43^.

The present findings also support an intrinsic vulnerability of cerebellum to blast-induced axonal injury, as no injury was observed in cerebral cortex, striatum, corpus callosum, or the hippocampal CA1 region. Consistent with a direct effect of blast, the cerebellar axonal injury was not lateralized and was apparent within 24 h of blast exposure. Lack of fluoro-jade B staining in the cerebellum at any time point corresponding to axonal silver staining excludes the alternative possibility that the axonal degeneration was secondary to death of the parent neurons. Though most studies of brain trauma omit evaluation of the cerebellum, several prior reports have identified cerebellar injury using a variety of blast exposure methods and outcome measures^37,44–47^. Head movement was not quantified in these studies, but those in which axonal injury was compared across multiple brain regions show general agreement with the present findings, i.e. blast-induced axonal injury identified most predominately (though not exclusively) in the cerebellum^37,46,47^. Studies of blast-exposed combat veterans similarly suggest evidence of cerebellar injury, as evidence by reduced glucose utilization^48^, diffusion tensor imaging tractography^49,50^, resting state connectivity^51^, and pathological findings^45^. In particular, Meabon et al. showed that mice subjected to single or multiple blast exposures developed cerebellar white matter injury and scattered Purkinje cell loss in an anatomical pattern similar to that evidenced by diffusion tensor imaging in blast-exposed veterans^50^.

Studies in which brain trauma was experimentally induced by methods other than blast also suggest that the cerebellum may be particularly sensitive to trauma. Where cerebellar pathology has been evaluated, injury has been identified not only with direct trauma, but also in models such as cortical fluid percussion, controlled cortical impact, and weight drop acceleration, in which the point of impact is anatomically remote from the cerebellum^52,53^. Likewise, the classic clinical / neuropathological study by Corsellis et al. of brains from retired boxers showed extensive cerebellar pathology, in addition to the more widely recognized supratentorial pathology^54^.

Results of the present studies showed, in addition to axonal injury in the cerebellum, a delayed but robust silver staining of axons in the visual system. However, the pattern of axonal damage to the visual system is indicative of degeneration secondary to trauma to the eye facing the blast wave, rather than to direct effect on the white matter tracts themselves. The eye was not shielded, and was thus vulnerable to damage caused by either the blast wave itself or, more likely, the associated blast wind. Axonal injury in the optic tracts has been previously described in rodent models of blast exposure and attributed to primary injury to the retina or proximal optic nerve^55–57^. We observed dense axonal injury in the optic tract contralateral to the blast-facing eye, the optic chiasm, and in the optic nerve ipsilateral to blast. (Note that roughly 90% of optic nerve fibers cross in the rat optic chiasm^24^). Notably, the association of activated microglia specifically with axons associated with the blast-facing eye suggest that the microglia activation, both there and in the cerebellum, is a response to the axonal injury rather than the cause of it.

### Limitations and conclusions

Animal models of blast injury vary considerably in intensity, animal shielding, animal orientation, head fixation, and other parameters, and extrapolation from studies performed in rodents using blast tubes to human injury induced by real-world blast exposure is limited by several well-recognized factors^58–63^. In particular, the physical dimensions of the rodent skull render it difficult to accurately mimic the blast-induced transcranial pressure gradients that can occur in the human. This is an unavoidable limitation of working with small animals. An additional unavoidable confound is that all animals were anesthetized. Gaseous anesthesia has potent neuroprotective properties, but it is unknown whether these agents also affect the mechanisms leading to axonal injury. A third consideration is that the reductionist approach employed here, in which effects of blast were largely isolated from effects of head movement and other secondary effects of blast exposure, does not account for the possibility that these injury mechanisms may have additive or even synergistic effects. Indeed, our data suggest this may be the case, as the cerebellar injury induced by blast appeared to be increased by concomitant lateral head movement. Of note, the intensity of blast wave striking the rat head was comparable to the intensity estimated to cause lethal pulmonary injury in humans^64^, so the lack of more extensive injury in the brains evaluated here cannot be attributed to blast intensity insufficient to mimic real-world exposure.

With these caveats, our results support the contentions that (1) blast per se can cause axonal damage independent of any head movement; (2) this injury is relatively minor in comparison to the other trauma mechanisms associated with blast exposure; and (3) that axons in cerebellar white matter are particularly vulnerable to direct blast injury. Additionally, the axonal degeneration observed in the visual system confirms prior observations indicating this as a secondary effect of ocular trauma.

## Supplementary Information

Supplementary Video S1.

Supplementary Video S2.

Supplementary Information.

## Data Availability

The data that support the findings of this study are available from the corresponding author on reasonable request.
