# Supplementary material for: Blast-induced axonal degeneration in the rat cerebellum in the absence of head movement
Source: Sci Rep. 2022 Jan 7;12:143. doi: 10.1038/s41598-021-03744-4 (PMC8741772; doi:10.1038/s41598-021-03744-4)

Supplementary Information

Blast-induced axonal degeneration in the rat cerebellum in the absence of head movement

#Robin Bishop, Seok Joon Won, Karen-Amanda Irvine, Jayinee Basu, Eric S. Rome, Raymond A. Swanson

#Robin Bishop and Seok Joon Won contributed equally to this work.

Supplemental Figure Legends

Supplemental Video S1. Video of rat blast exposure with head-movement holster.

Supplemental Video S2. Video of rat blast exposure with head-fixed holster. Representative of six recordings, all showing no detectable linear or rotational head movement.

Supplemental Figure S1. Absence of sliver-stained nerve fibers in cerebral cortex and striatum. Rectangles in low power views (top) show regions photographed. Silver staining in (a) cerebral cortex and (b) striatum showed no labeled fibers. Scale bar = 30 µm. Images are representative of n = 3 rats treated at each time point after exposure to blast with head movement allowed.

Supplemental Figure S2. Absence of silver-stained nerve fibers in hippocampus and corpus callosum. Rectangles in low power views (top) show regions photographed. Silver staining in (a) hippocampal CA1 and (b) corpus callosum showed no labeled fibers. Scale bar = 30 µm. Images are representative of n = 3 rats treated at each time point after exposure to blast with head movement allowed.

Supplemental Figure S3. Morphological and APP markers of axonal injury. All images are from superficial cerebellar white matter, with the cellular layer at the bottom of the image. Rats were exposed to blast with no head movement, and brains harvested 3 days later. (a) High magnification view of silver staining shows focal varicosities in some fibers (red arrowheads). (b) Immunostaining for neurofilament-H shows axonal varicosities in blast exposed brains (top, black arrowheads), but not in controls (bottom). (c) Immunostaining for amyloid precursor protein (APP) shows scattered foci of accumulation in the blast-exposed brains (top, black arrowheads), but not in the sham blast controls (bottom). Images are representative of sections taken from 3 brains under each condition. Scale bar = 30 µm.

Supplemental Figure S1

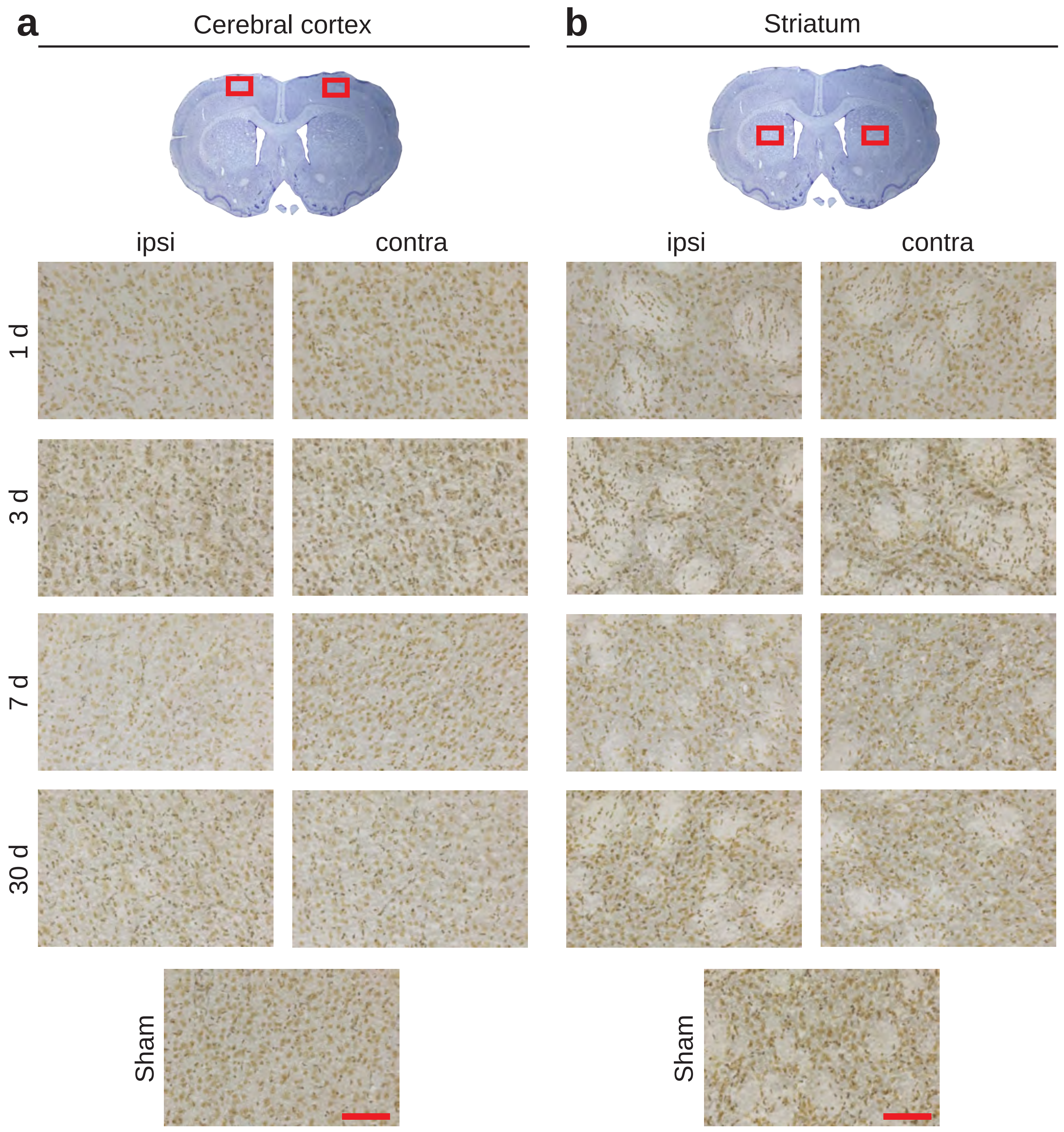

Supplemental Figure S2

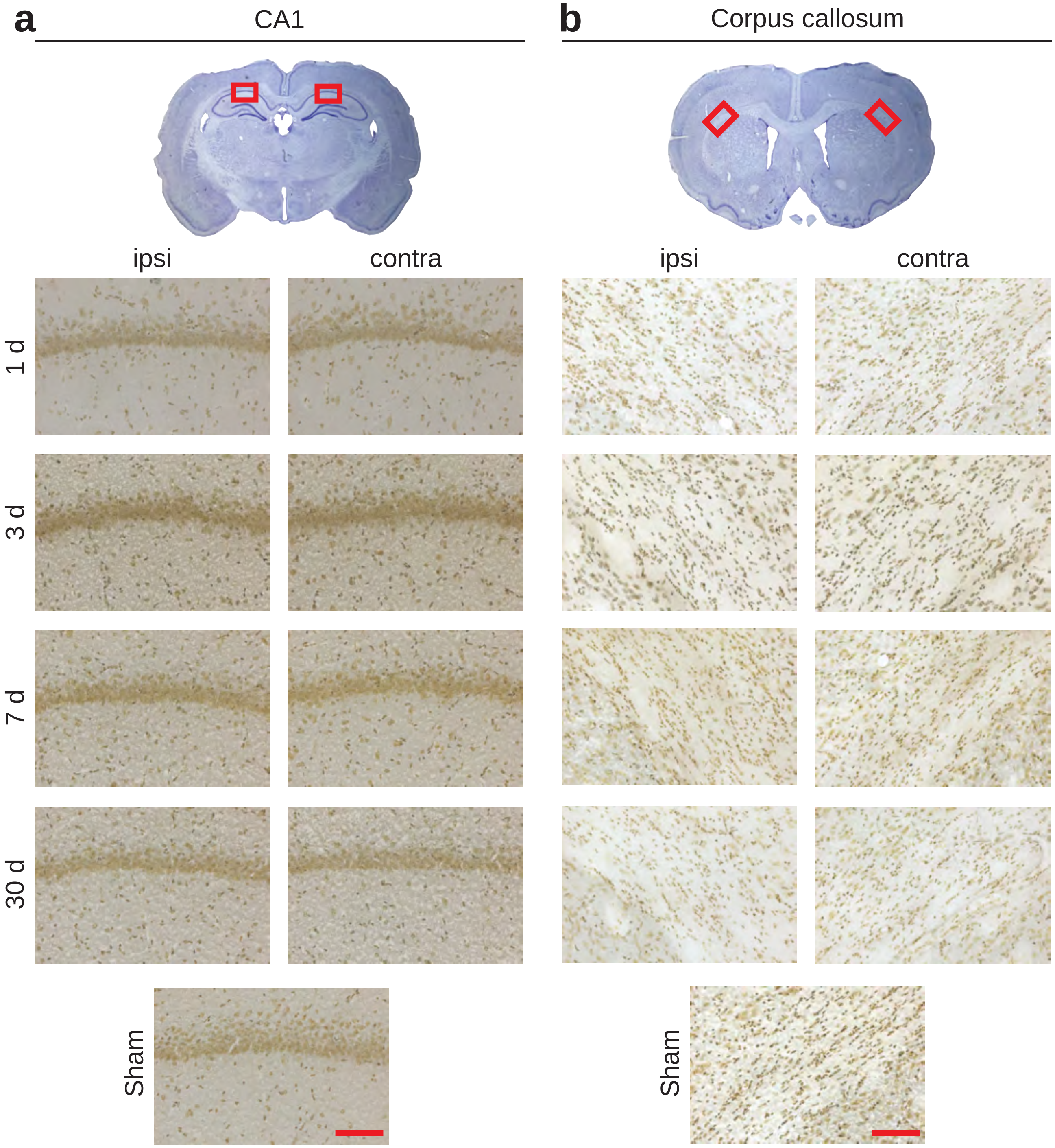

Supplemental Figure S3

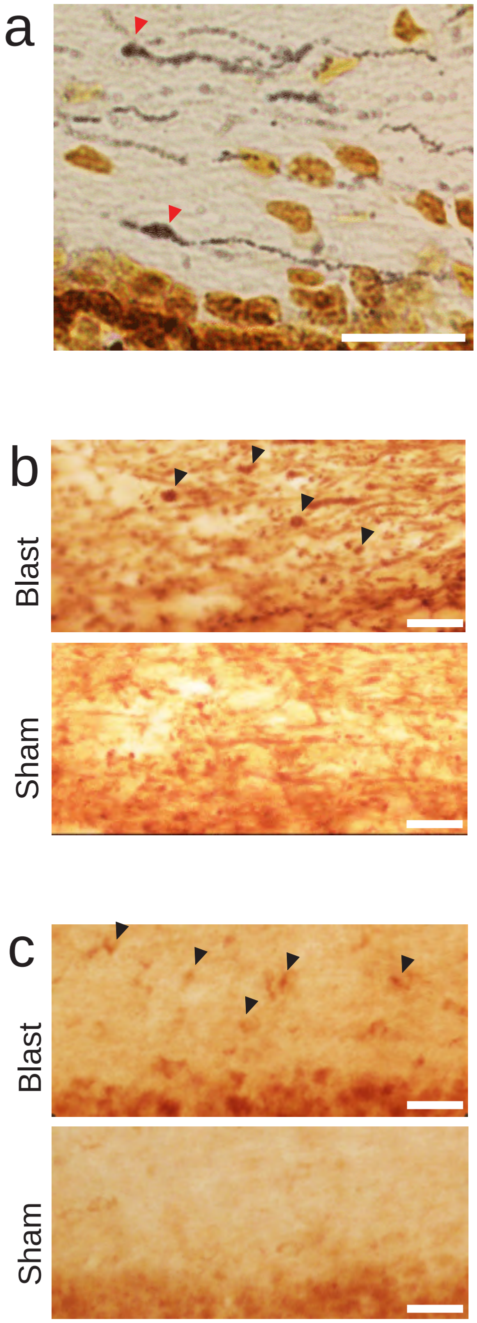

Supplement: Supplementary file 3 — Supplementary Information. [file 41598_2021_3744_MOESM3_ESM.pdf]
